# Moisture mitigation using a vented liner and a vented socket system for individuals with transfemoral amputation

**DOI:** 10.1038/s41598-023-43572-2

**Published:** 2023-10-02

**Authors:** Surya C. Gnyawali, Jeffrey A. Denune, Bryce Hockman, Jóna Valgerður Kristjánsdóttir, Margrét Sól Ragnarsdóttir, Lava R. Timsina, Subhadip Ghatak, Knut Lechler, Chandan K. Sen, Sashwati Roy

**Affiliations:** 1 McGowan Institute for Regenerative Medicine (MIRM), Department of Surgery, University of Pittsburgh School of Medicine, Pittsburgh, PA USA; 2 Indiana Centre for Regenerative Medicine and Engineering (ICRME), Indiana University Health Comprehensive Wound Centre, Indiana University School of Medicine, Indianapolis, IN USA; 3 Össur Ehf., R&D, Medical Office, Reykjavik, Iceland

**Keywords:** Biotechnology, Anatomy, Health occupations, Medical research

## Abstract

Sweating and heat buildup at the skin-liner interface is a major challenge for persons with limb loss. Liners made of heat-non-conducting materials may cause sweating of the residual limb and may result in liners slipping off the skin surface especially on a warm day or during high activity, causing skin breakdown and affecting limb health. To address this, we evaluated the efficacy of the vented liner-socket system (VS, Össur) compared to Seal-In silicone liner and non-vented socket (nVS, Össur) in reducing relative humidity (RH) during increased sweat. Nine individuals with limb loss using nVS were randomized to VS or nVS and asked for activity in a 20-min treadmill walk. RH was significantly attenuated (*p* = 0.0002) and perceived sweating, as reported by prosthesis users, improved (*p* = 0.028) with VS, patient-reported comprehensive lower limb amputee socket survey (CLASS) outcomes to determine the suspension, stability, and comfort were not significantly different between VS and nVS. There are limited rigorous scientific studies that clearly provide evidence-based guidelines to the prosthetist in the selection of liners from numerous available options. The present study is innovative in clearly establishing objective measures for assessing humidity and temperatures at the skin-liner interface while performing activity. As shown by the measured data and perceived sweat scores provided by the subjects based on their daily experience, this study provided clear evidence establishing relative humidity at the skin-liner interface is reduced with the use of a vented liner-socket system when compared to a similar non-vented system.

## Introduction

In 2005, the number of person living with the major lower extremity amputation was 1.6 million, this number is projected to increase by approximately 2.2 fold by 2050^1^ . Transfemoral amputation (TF) accounts for approximately 40% of all lower-limb amputations in the USA^2^. Prosthetic satisfaction is central for optimizing the use of the prosthesis, preventing rejection, and increasing compliance with the medical regimen. Optimal socket design, fit, and interface materials are critical for successful prosthetic use for individuals with lower extremity limb amputations. Sub-optimal fit and comfort of the prosthetic system are known to cause rejection of the prosthesis and preference toward other assistive devices such as wheelchairs^3^. However, prosthesis use provides a realistic appearance that helps users hide the sense of limb loss compared to wheelchairs that are more easily noticeable^4^.

Sweat (moisture) and heat build-up at the skin-liner interface remains a major challenge that reduces the quality of life for amputees. According to a systematic review of associated factors and questionnaires, it has been documented that heat and sweat are leading complaints that result in a reduced quality of life^5^. The confinement of the residual limb in a warm and moist environment for a prolonged period of time can result in bacterial infections and allergic reactions^5^. Bacterial infection-related complications occur in 20–41% of amputees^6^. Blisters and ulcers, the most common skin ailment related to prosthesis use, often begin as abrasions caused by friction between the prosthetic liner and the residual limb^6^. A prosthetic liner is an integral part of overall suspension system and provides cushioning thus, improvement in the normal and shear stress distribution around the limb^7^. Furthermore, the sweat buildup inside the liner may result in the liner sliding on the skin thereby affecting the suspension of the prosthetic leg^8^. Breakdown of the epidermal skin barrier function is one of the most predisposing factors for the development of skin allergic diseases thereby complicating residual limb health^9–11^.

Elevated skin temperature and sweat build-up are commonly associated with wearing and using prosthetic liners that are primarily made of occlusive materials that retain heat^12^. Prolonged use of such occlusive material results in sweating of the residual limb^13^. Measurement of in-socket residual limb sweating, or moisture build up at skin and liner interface during activity is critical to design strategies to manage moisture at skin-liner interface. Traditional methods of measuring the amount of sweat via gravimetry where the sweat quantitatively recorded by wiping the residual limb and internal liner surface with a laboratory towel and placing the towel in a sealed plastic bag and weighing on a digital scale^14^. However, this method is not able to perform sweat measurements in real time and with high precision.

**Figure 1:**
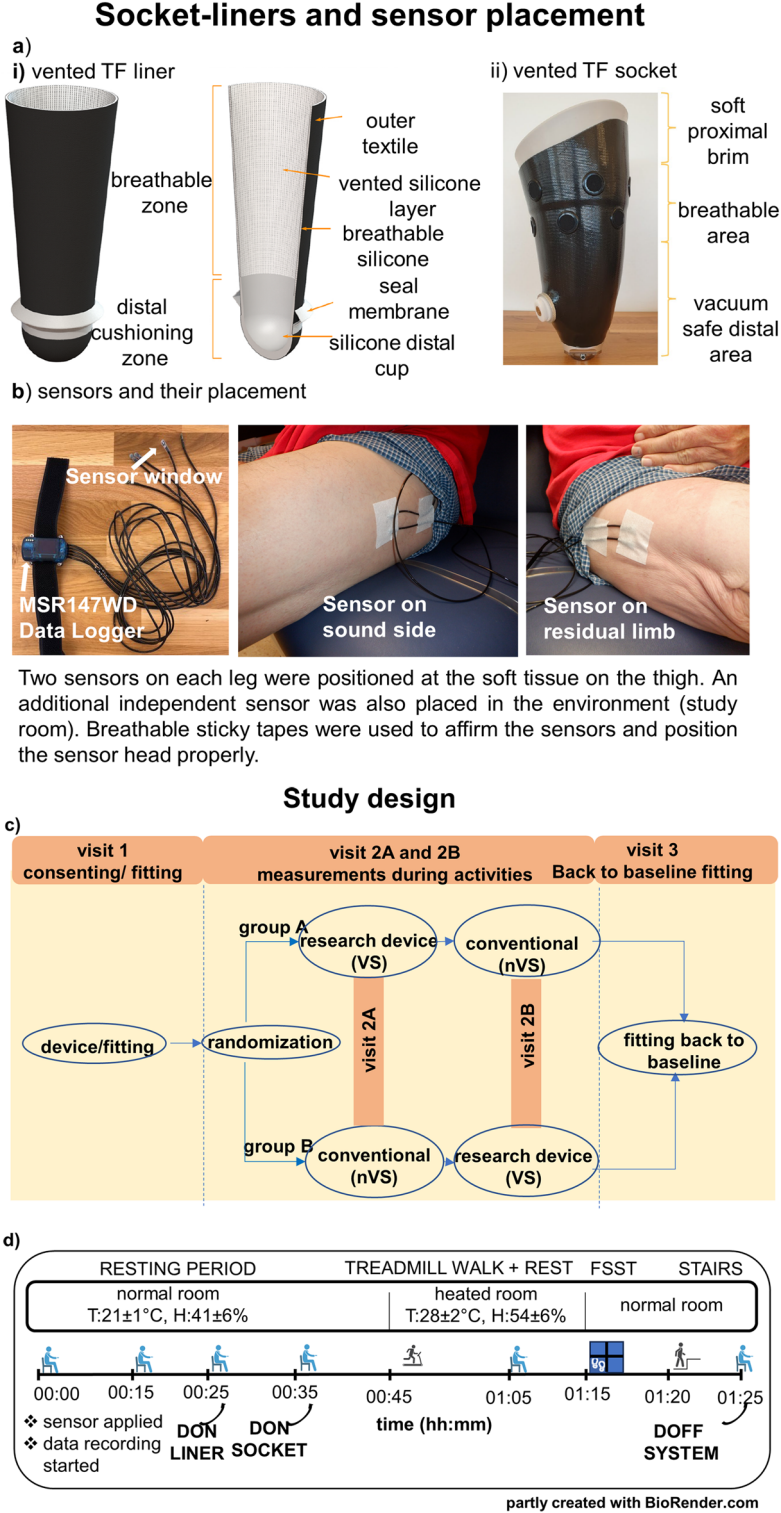
Components of vented liner-socket system used in the study.** (1-1a-i)** The full outer and cross-sectional view of the vented silicon liner,** (1-1a-ii)** the vented TF socket components, **(1-1b)** MSR data logger, a device that records relative humidity and temperature from limbs (left). Twin sensors were placed on the sound limb (center panel) and on the residual limb (right panel). The sensors were placed on limbs and secured with breathable sticky medical tape ensuring the sensor window facing away from the skin to prevent saturation of the sensor. Study design and activities. (**1-2c**) Visit 1- patient consenting, conventional and investigational socket fitting and randomization was performed. Visit 2A–2B-included crossover design from randomized vented liner/vented socket solution (VS) or non-vented liner socket solution (nVS) groups followed by fitting back to conventional system in visit 3. (**1-2d**) Schematic timeline of measurements of temperature and relative humidity during activities: resting, liner and socket donning, treadmill walk, four square step tests (FSST), and stair walk. The study room was maintained at temperature (T): 21 °C ± 1 °C, humidity (H): 41% ± 6%. The activities (red shaded) were performed in an environmentally controlled room maintained at T: 28 °C ± 2 °C, H: 54% ± 6%.

There are limited rigorous scientific studies that clearly provide evidenced-based guidelines to the prosthetist in selection of liners from numerous available options. A need to develop objective metrics to evaluate a liner along with scoring for patient satisfaction has been identified^15^. To address this gap, we propose a clinical study that will test the hypothesis that vented liner/ socket system as compared to a non-vented socket system will provide effective mitigating of moisture at the skin-liner interface during increased sweating in warmer temperatures while performing activity^16^. This clinical study will test a novel vented liner/vented socket system (Fig. 1a) specifically developed to alleviate moisture build-up at the socket/limb interface^16^. The vented liner consists of an inbuilt mesh material to allow vertical and horizontal air flow intended to be triggered by intermittent loading and unloading of the interface as well as allowing moisture to travel through the liner and out of the vented socket.

## Methods

The study protocol was approved by the Institutional Review Board of Indiana University (IU) IRB #12377 and Advarra (Pro00055181). All methods were performed in accordance with the Declaration of Helsinki. All study subjects signed informed consent prior to participation.

### Subjects recruitment and eligibility criteria

All participants were identified from the prosthetics clinic at the SRT National Prosthetic Center, Indianapolis, USA. Potential subjects were initially screened for their eligibility to participate in the study. The study team then verified if an identified subject was interested in participating in the study. If interest was expressed at this point, the study team made an appointment for signing the informed consent form followed by evaluation by a certified study prosthetist confirming all eligibility criteria to participate in the study were met.

The subjects were deemed eligible to participate in the study if the following inclusion and exclusion criteria were met: (1) subjects of age more than 18 years; (2) body weight in the range of 50–136 kg; (3) no cognitive impairment; (4) unilateral or bi-lateral transfemoral amputation; (5) able to walk on a treadmill for 20 min; (6) using Seal-In Silicone Liner and Direct Socket (Össur). The subjects were using the same liner profile and the same socket material as for direct socket. The AeroFit is a variant of the direct socket transfemoral (DSTF)^17^ and the AeroFit liner is a variant of the Seal-In liner with the same profile. (7) Met predefined criteria for minimum stump length, liner size and residual limb shape; (8) were willing and able to participate in the study and follow the protocol; and (9) and were experienced prosthetic users for more than 3 months. The subjects were excluded if they were participating in any other study for the duration of the present study.

### Study design

**Figure 2:**
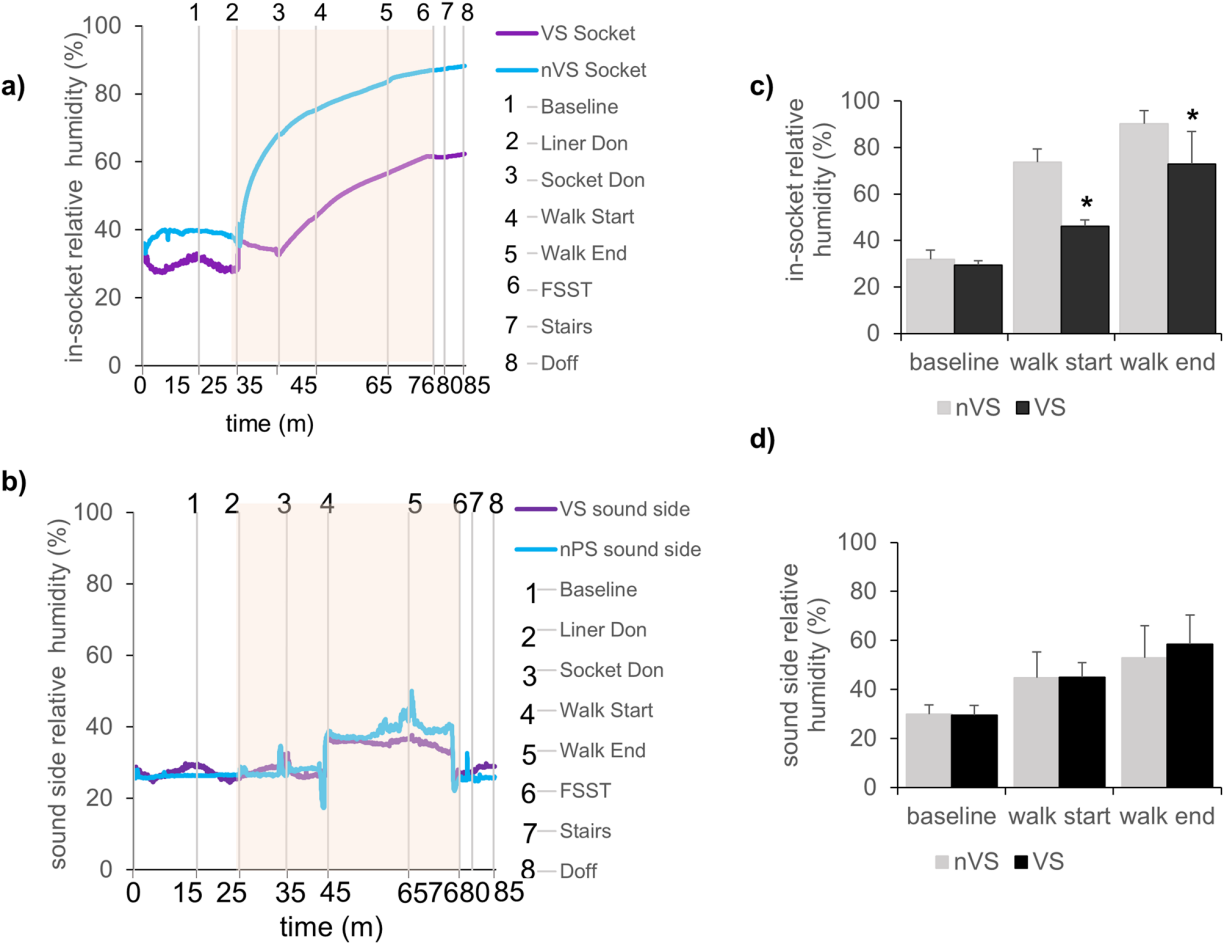
Reduction in relative humidity measurements on the skin liner interface following activity.** (a, b)** Representative relative humidity plot **(a)** from residual or **(b)** sound limb of the same individual. Blue line present data from nVS while the purple line presents the VS system. Gray vertical lines show the time events during study. 1-baseline, 2-liner don, 3-socket don, 4-walk start, 5-walk end and resting, 6-four square step test (FSST), 7-walking on stairs, and 8-doff the socket-liner obtained for data set for 1 h and 25 min. Data presented mean ± SD, n = 9, *p < 0.05. nVS = non-vented liner socket solution, VS = vented liner/vented socket solution.** (c, d)** Bar graphs showing **(c)** residual limb or **(d)** sound side humidity in all patient visits. Bar graphs data showing mean environmental humidity measured on all patients during treadmill walk activity compared to baseline. Data presented mean ± SD, n = 9, *p < 0.05. nVS = non-vented liner socket solution, VS = vented liner socket solution**.**

The clinical pilot study was a randomized, controlled, open label, cross-over study design with the endpoints of relative humidity (RH), perceived sweat, stability, comfort, and suspension. All participants were fitted to the vented investigational device at SRT National Prosthetic Center, Indianapolis, USA. All other study related activities occurred at Indiana University School of Medicine, Indianapolis, IN. After a minimum of two weeks of adaptation time, participants were randomized to two arms of this study: (a) Interventional device arm: a vented investigational device (AeroFit™ socket and liner), Össur (VS); and (b) SoC or comparator device arm: Seal-In® silicone liner and the AeroFit Socket with sealed vents, Össur (nVS). The relative humidity and temperature sensors were placed at the soft tissue on residual limb thigh and sound leg thigh (Fig. 1-1a, 1-1b). One additional sensor was placed on the environment. Breathable self-adhesive tape (Hypafix, BSN Medical, Germany) was used to affix the sensors and positioned the sensor head properly. RH and temperature in the skin/liner interface were measured throughout the entire study procedures as presented (Fig. 1-2c, 1-2d). The procedure started in a regular temperature room (21 °C ± 2 degrees) with a 10-min resting period with no prosthesis on for a baseline measurement. Then another 10-min resting period with only the liner on and 10 min with the liner and socket on. After resting, the subjects moved to a heated (28 °C ± 2 degrees) and humidity (42% ± 2%) controlled room where they walked at a self-selected speed on a treadmill for 20 min wearing the MSR Data Logger and sensor system (Fig. 1-1b). After 10 min resting, subjects left the heated room, entered the normal temperature room, performed the FSST and walked stairs. At last, patient-reported outcomes (perceived sweat and CLASS^18^) were administered (Fig. 1-2d). Measures were taken to ensure a repeated and controlled environment for both visits to each subject. This included having the user come in for both visits at the same time of day, wearing the same or similar clothing (recommended to wear shorts) and setting the same treadmill speed for both visits. A table summarizing the study visits and procedures has also been provided (Fig. 1-2c).

### In-socket humidity and temperature measurement

Relative humidity and temperature data were acquired using an MSR147WD wireless data logger system with sensors (MSR Electronics GmbH, Seuzach, Switzerland) on the skin (Modular Signal Recorder) held in place as shown in Fig. 1-1b. After completion of data collection, the raw data was transferred to the computer for further analyses using MSR software. Data were acquired continuously during baseline and throughout the activities (Fig. 1-2d). The MSR software provides a spreadsheet of percent RH and temperature in degrees Celsius (^°^C). The RH and temperature data from two sensors on each leg were averaged. The data from temperature and humidity sensors for the study room (environment) was also recorded.

### Perceived sweat score and CLASS

Subjects rated their perceived sweat during activity on a scale from 1–10 where 1 is an extreme amount of sweat, and 10 is no sweat at all. A higher score corresponds to less perceived sweat on the skin. The Comprehensive Lower Limb Amputee Socket Survey (CLASS) is a self-report measure of prosthetic socket satisfaction that quantifies suspension, stability, comfort^19^. The scale is 1–4, from strongly disagree to strongly agree, 0 is not applicable.

### Quantification of the area under the curve using trapezoid rule

In numerical analysis and scientific computing, the trapezoidal rule is a numerical method to solve ordinary differential equations derived from the trapezoid rule for computing integrals^20^. The trapezoidal rule is an implicit second-order method that approximates higher order terms of the function^20^. The trapezoid rule was applied to accurately determine the area under the time-humidity curve (AUC) via a locally written MATLAB® code. The AUC of the residual was normalized using the corresponding values from the sound limb for each subject using the following equation

1 $$VS_{norm} = \frac{{VS_{RL} }}{{VS_{SL} }}$$

2 $$nVS_{norm} = \frac{{nVS_{RL} }}{{nVS_{SL} }}$$

where RL = residual limb and SL = sound limb and VS = vented liner/vented socket; nVS = non-vented liner/socket.

The measurement of percent reduction of relative humidity by VS was calculated as:

3 $$AUC_{norm} = \frac{{(AUC(nVS_{norm } ) - AUC(VS_{norm} ))*100}}{{AUC(nVS_{norm} )}}$$

Using the trapezoidal rule algorithm, AUC was calculated from the relative humidity-time curve during a 50 min period of all activities performed as a part of the study (from liner donning to exit heated room) (Fig. 4a, b). The plot in Fig. 4c represents the % reduction in nVS compared to VS derived from the Eq. (3).

### Statistical analysis

Descriptive statistics were used to describe the study population using mean, standard deviation for continuous variables and frequency and percentages for categorical variables. The normality of the continuous variables was checked and confirmed using Shapiro–Wilk tests. To account for dependencies of the measured metrices, we used paired t-tests to compare the stability, suspension, and comfort scores using Comprehensive Lower Limb Amputee Socket Survey (CLASS), perceived sweat, and relative humidity AUC between nVS and VS systems. For the 2 × 2 crossover design, using Analysis of Variance (ANOVA) we evaluated inter- and intra-subject variabilities, sequence, period, and treatment effects if no carryover effects exist. One-tailed t-test was performed because the primary outcome hypothesis tested was effect on effective mitigation/decrease of sweat within liner. All statistical testing and analysis were done using Stata/MP 16.1.

## Results

Nine participants completed all protocol procedures and used for data analysis. A total of 13 adult human subjects (12 male and 1 female) were consented and enrolled in this study. One participant was only part of the pilot run and not the formal data collection phase. Two subjects were withdrawn from study. One patient was excluded from data analysis due to a protocol deviation. Therefore, data analysis was performed on a total of 9 subjects, 8 males and 1 female with mean age of 46 ± 14 years. Reasons for amputation were trauma and infection. Time since amputation varied from 15 years to under a year. Considering mobility, eight subjects were a K3 level, and one was a K2 level activity (Table 1). Two adverse events (AE) were reported during the study period. One of these was determined to be unrelated to the study while the other AE was related to developing skin irritation with the use of investigational device, the AE resolved and the participation for this subject (ID: AF-10) was terminated from the study.

**Table 1:** Patient demographics summary and prosthetics foot/knee details.

Population characteristics	In sample, n (%)
Age in years (mean ± SD)	46.5 ± 14.03
Sex n (%)	
Male	8 (88.89)
Female	1 (11.11)
Activity level n (%)	
Level 3 (K3)	8 (89.89)
Level 2 (K2)	1 (11.11)
Cause of amputation n (%)	
Traumatic	8 (88.89)
Infection	1 (11.11)
Prosthetic foot n (%)	
Freedom renegade	1 (11.11)
Pro-Flex LP torsion	1 (11.11)
Pro-Flex XC torsion	2 (22.22)
Pro-Flex Pivot	1 (11.11)
Freedom Kinterra 2.0	1 (11.11)
Unknown	4 (44.44)
Prosthetic knee n (%)	
Freedom Plie 2	1 (11.11)
Ottobock C-Leg	1 (11.11)
Power Knee	1 (11.11)
Rheo Knee	5 (55.56)
Rheo Knee XC	1 (11.11)

Patients with an average age of 46.5 (mean ± SD, 46.5 ± 14.03) participated in the study. Gender: 8/9 were male and 1/9 was female. Reason for amputation: eight patients with traumatic amputation and one infection. Data presented as patient number, n (%).

### The vented system (VS) significantly attenuated relative humidity (RH) and no change in temperature on the skin liner interface following activity in a climate-controlled room

Each session for determination of RH lasted 1 h and 25 min and occurred in an ambient temperature room (T:21 °C ± 1 °C, H:41% ± 2%) followed by performing activities in a heated room (T:30 °C ± 1 °C, H:54% ± 6%) then post activity return-back to the ambient temperature room (Fig. 1d). The data from residual limb and sound side limb have been presented as continuous recording of RH as a function of time (Fig. 2a, b). There was no meaningful change in the baseline humidity both on residual limb (Fig. 2a) and sound side (Fig. 2b) as shown by the representative plots. However, during activity the humidity significantly elevated compared to baseline (Fig. 2c) on the residual limb side as compared to sound side (Fig. 2d). The average RH during activity significantly attenuated in residual limbs with the use of VS as compared to nVS (Fig. 2c).

**Figure 3:**
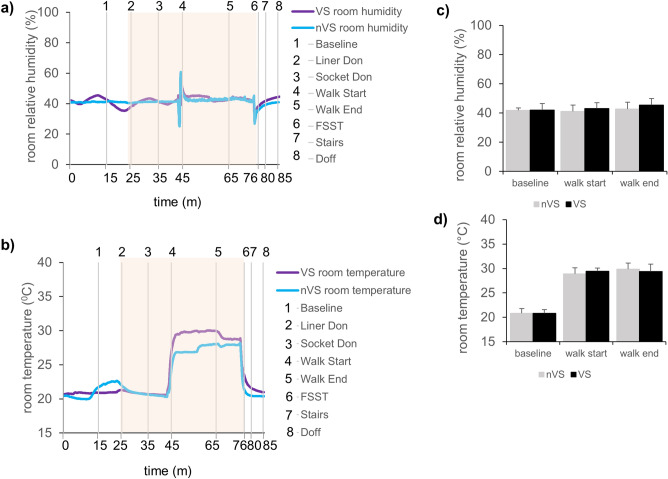
Relative humidity and temperature of environment in the study room.** (a, b)** Representative relative humidity/temperature data plots **(a)** environmental humidity or **(b)** environmental temperature. Blue lines represent data from nVS while purple lines represent the VS system. Gray vertical lines show the time events during study. 1-baseline, 2-liner don, 3-socket don, 4-walk start, 5-walk end and resting, 6- four square step test (FSST), 7- walking on stairs, and 8-doff the socket-liner obtained for data set for 1 h and 25 min. Light orange background on the representative plots indicates the activity time zone.** (c, d)** Bar graph showing **(c)** environmental humidity or **(d)** environmental temperature in all patient visits. Bar graph data showing mean environmental humidity measured on all patients during treadmill walk activity compared to baseline. Data presented mean ± SD, n = 9, *p < 0.05. nVS = non-vented liner socket solution, VS = vented liner socket solution**.**

The RH environmental data collected simultaneously with the RH from skin-liner interface confirmed that the reduction in RH at the skin interface was not due to changes in environmental conditions. The conditions were comparable in both VS and nVS groups (Fig. 3). Representative continuous recordings and average of environmental relative humidity (Fig. 3a, b) and temperature (Fig. 3c, d) of the study room has been presented for consistency.

**Figure 4:**
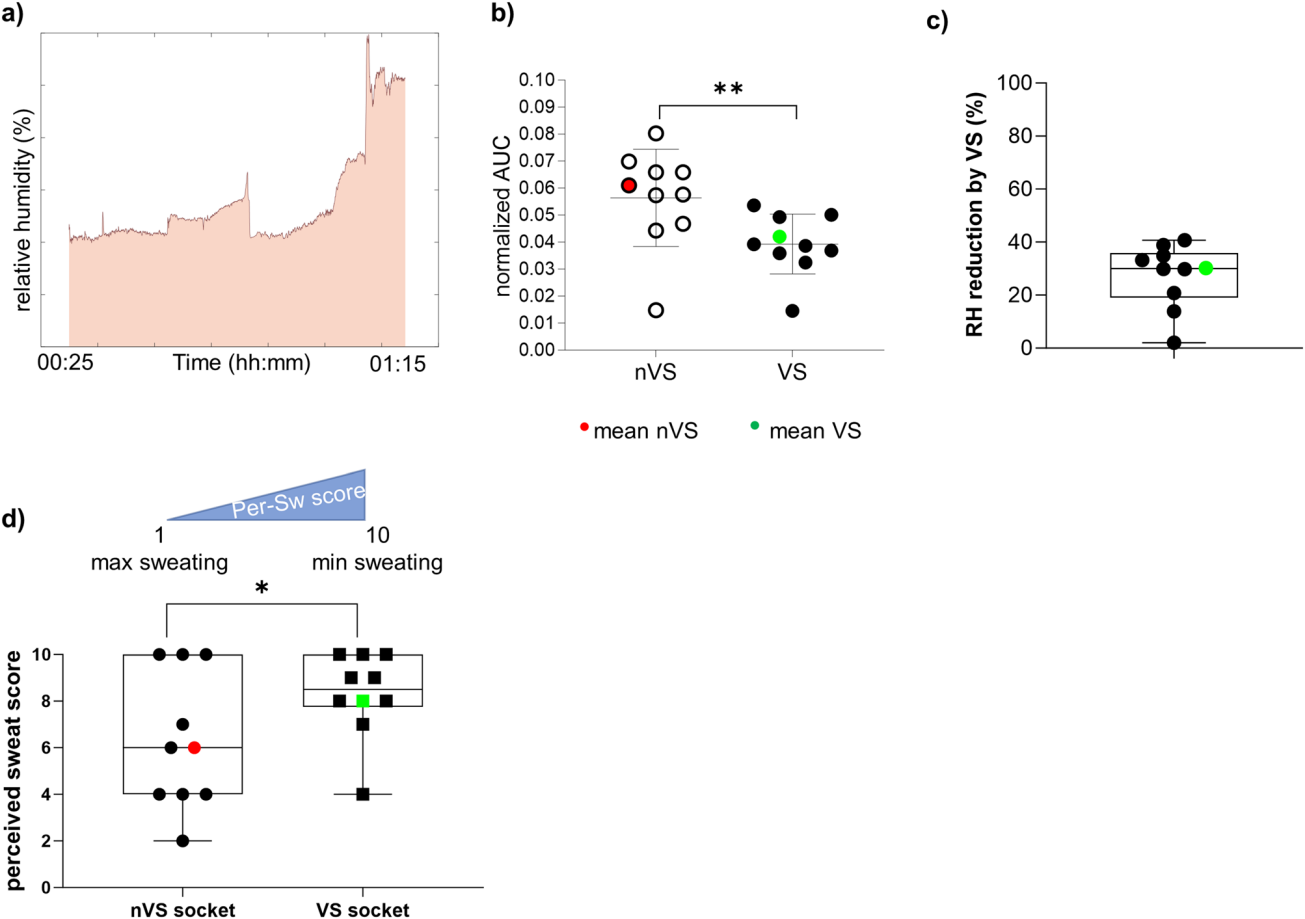
The reduction in mean relative humidity by VS in the residual limb normalized to sound limb.** (a)** The representative area-under-the curve (AUC) used to measure changes in overall levels of relative humidity (RH) for the duration of all activities. The AUC was calculated using locally written MATLAB® code. Relative humidity data from VS or nVS sockets were normalized with the respective sound side limb data. The shaded area represents AUC during the activity time (50 min) of the study. (**b**) The normalized RH from all subjects (mean, red or green) in nVS or VS group data are color-coded. Data from (**b**) using Eq. (3) were used to derive the plot (**c**) which is a percent RH reduction by VS. Data presented as mean ± SD, n = 9, ** *p* = 0.0002. nVS = non-vented liner socket solution, VS = vented liner socket solution. The perceived sweat score from patient survey demonstrates significantly lower sweating in VS group as compared to nVS (**c**). Subjects rated their perceived sweat during activity on a scale from 1 to 10 where 1 is an extreme amount of sweat, and 10 is no sweat at all. Data presented as box blot of n = 9, *p = 0.038). nVS = non-vented liner socket solution, VS = vented liner socket solution, Per-Sw score = perceived sweat score.

To further investigate the humidity change during activity, AUC was computed and the representative relative humidity-time graph highlighting the region considered is shown (Fig. 4a). The area under the humidity-time curve, a calculus-based metric, was analyzed from the raw humidity data normalized with sound side (Fig. 4b). Then AUC data from Fig. 4b was used to calculate the percent reduction in humidity using the Eq. (3) (Fig. 4c). The results showed that the mean AUC-based percent reduction of humidity in VS arm was 30 ± 12.50% when compared to nVS.

Perceived sweat was one of the secondary outcomes. A paired, one-sample, two-tailed t-test was used to compare perceived sweat ratings between the VS and nVS (*p* = 0.028, 95% CI [0.12, 3.88], Fig. 4d).

**Figure 5:**
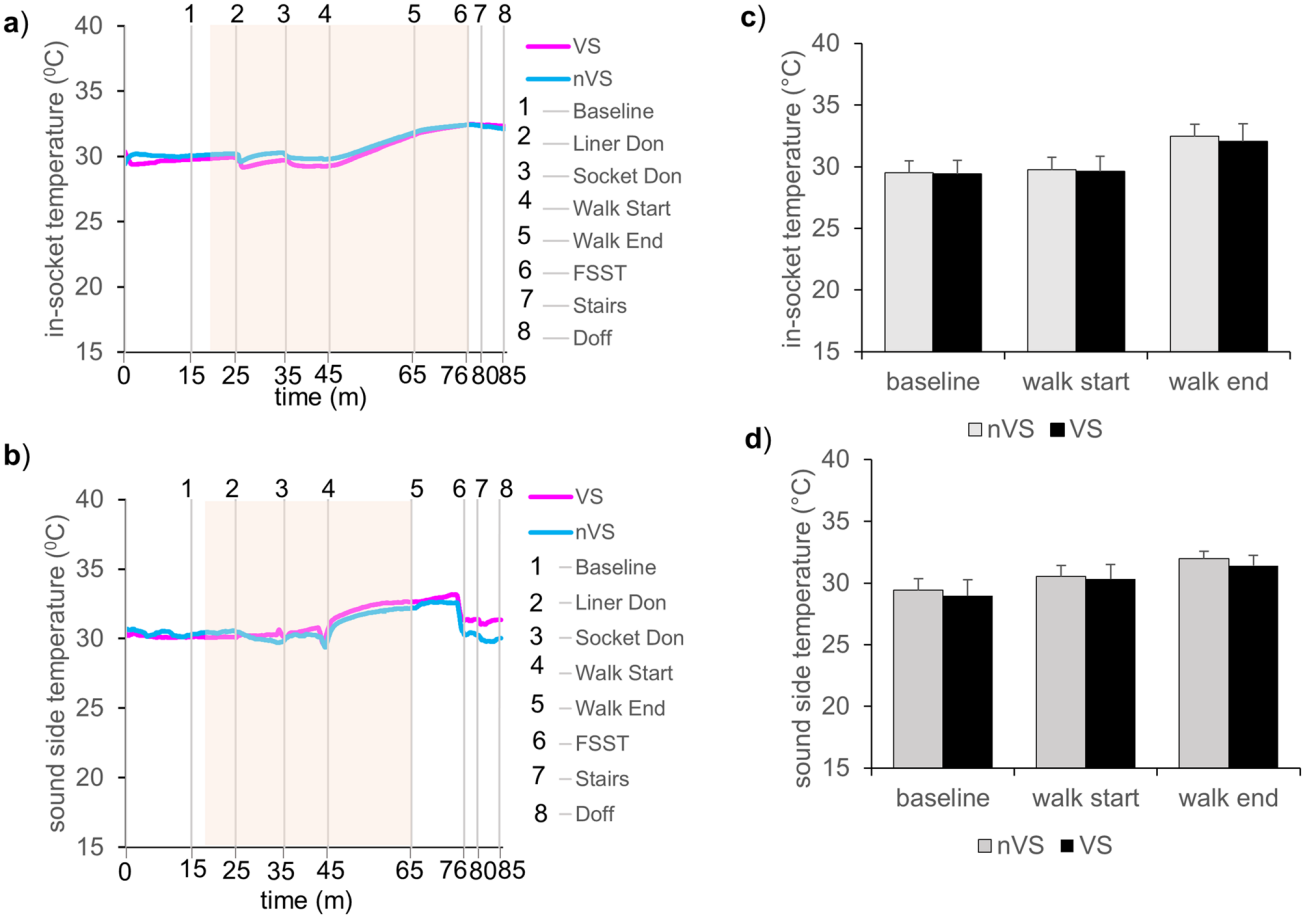
Temperature changes during activity compared to baseline. Representative temperature plots **(a)** in-socket temperature of the residual limb or sound side temperature **(b)**. Blue line represents data from nVS while pink line represents the VS system. Gray vertical lines show the time events during study. 1-baseline, 2-liner don, 3-socket don, 4-walk start, 5-walk end and resting, 6- four square step test (FSST), 7- walking on stairs, and 8-doff the socket-liner obtained for data set for 1 h and 25 min. Light orange background on the representative plots indicates the activity time zone. Bar graphs showing in-socket temperature **(c)** or sound side temperature **(d**) of all patients**.** Data showing mean temperature measured on all patients during treadmill walk activity compared to baseline. Data presented mean ± SD, n = 9, *p < 0.05. nVS = non-vented socket solution, VS = vented socket solution.

During activity, an increase in temperatures within sockets has been reported to result in discomfort^21^. Current study showed no significant increase in the in-socket temperatures on the residual limb (nVS vs VS) or skin temperature in the sound limb side suggesting adequate thermoregulatory processes keeping the skin temperatures unchanged. Temperature change of nVS and VS systems compared between the in-socket temperature of the residual limb and sound side during study time are shown (Fig. 5a–d) with no significant difference in temperature change between VS and nVS arms.

### The investigational VS did not significantly affect perceived stability, suspension, or comfort

CLASS stability subsection was used to evaluate perceived stability^19^. One-tailed Wilcoxon-signed rank test was used to determine whether the median of CLASS stability score of VS compared to nVS, was no worse. The significance level was set at *p* < 0.0167 and margin of non-inferiority (d) was −7% (-0.07). The median was 0.063 and 98.33% CI was (-0.031, Inf). The CI does cross intervention difference of 0 but not the margin of noninferiority. The VS and nVS are not different with respect to perceived stability during increased sweating. A table summarizing and mean ± SD and statistical significance of all endpoints have been presented (Table 2).

**Table 2:** Summary of statistical interpretation of all study endpoints.

Indicators	nVS	VS	p-value
Humidity (mean, SD)	0.015 ± 0.007	0.011 ± 0.005	0.0005
Perceived sweat (mean, SD)	7.220 ± 2.818	8.44 ± 2.006	0.0280
Comprehensive lower limb amputee socket survey (CLASS)			
Stability (mean, SD)	87.500 ± 13.970	86.8 ± 14.470	0.6595
Suspension (mean, SD)	84.720 ± 14.010	86.8 ± 16.660	0.2619
Comfort (mean, SD)	84.720 ± 17.708	84.72 ± 16.568	0.5000
Total CLASS (mean, SD)	85.640 ± 12.940	86.11 ± 14.430	0.3729

The analyses were done according to the hypotheses of sweat (B1), stability (C1), suspension (C2), comfort (C3), and the area under the curve (A1) as proposed in the study protocol. The comparison was done between the vented test socket and the SoC socket. For the 2 × 2 crossover design, using analysis of variance (ANOVA) we evaluated inter-, and intra-subject variabilities, sequence, period, and treatment effects considering if no carryover effects exist.

## Discussion

The prosthetic liners are essential and a significant component of socket fitting and usage^22^. The liners act as an interface between rigid socket and soft tissue^17^. The liners have two major functions, to protect the soft tissue of the residual and provide a good coupling with the socket-prosthesis^2^. Therefore, it is critical for a prosthetist to carefully consider the individual need for a patient and select an adequate liner that will perform both functions (soft tissue protection and coupling) well to provide a comfortable experience to the user while minimizes long-term problems associated with skin such as dermatitis and ulceration. The present study is innovative in clearly establishing objective measures in assessing humidity and temperatures at the skin-liner interface at baseline and while performing activity. This study provided maiden evidence that relative humidity at skin-liner interface is reduced with the use of vented liner-vented socket system when compared to a similar non-vented system. Such reduction in relative humidity did not change temperatures or compromise socket fit, comfort or suspension. Such reduction in relative humidity did not change temperatures or compromise socket fit, comfort or suspension.

From clinical relevance perspective, the prosthetic liners may be categorized based on their elasticity, conductivity and coefficient of friction (CoF) properties^23^. Based on a recent review, there are over 70 commercial liner products for transtibial amputee in the market^24^. The conventional use of silicone liners is primarily for comfort and impact absorption, while providing a close fit and suspension^25–27^. However, these liners are occlusive and may create warm, moist environments that are ideal for microbes growth^26^ and lead to uncomfortable socket fits including slip off from the limb skin surface causing accidents and injuries^12,28^. Inappropriate suspension can cause shear forces which can lead to skin breakdown compromising the barrier function of residual limb health^28^. Over seventy-five percent of the patient population with lower-limb prosthetics have skin problems^29,30^. An ideal liner should be able to not only provide cushion for the transfer of loads from the prosthetic socket to the residual limb but also efficiently manage moisture at the skin liner interface while providing a comfortable socket fit.

Perceived thermal discomfort within the liner during use of prosthetics is a commonly reported outcome by the users. There is a focus on development of new prosthetic technology to address thermal discomfort with the use of liners^31^. Using thermistor sensors, a study investigated if activity affected skin temperatures. Walking on a treadmill caused a significant (3.1 °C) increase in skin temperatures inside the prosthesis. Rest was insufficient to provide thermal relief without doffing the prosthesis^31^. In recent years, phase change material (PCM) technology has been adopted for the use in prosthesis liners. These promise to improve temperature control and, consequently, reduce sweating^32^. A randomized double-blind cross-over study was conducted to evaluate the use of phase-change material-based temperature-control liners have clinically meaningful effects^32^. Of the 42 enrolled participants, only 50% completed the protocol. The high attrition was in large part because of the COVID-19 pandemic. The findings indicate that the temperature control liners were, by trend, associated with better prosthesis utilization. The found effects did not reach the level of statistical significance, which is likely a result of the unduly reduced sample size. Our study also clearly shows that there are no significant changes in skin temperatures at the skin-liner interface, suggesting a clear role of thermoregulation in maintaining the skin temperature^33^. Our data and other studies question the significance of liner designs that only consider dissipation of heat at the interface.

Despite an effective moisture reduction in the skin-liner interface, the current study did not find any differences in the CLASS score as rated by the subjects, which may be because of inadequately powered study to determine if moisture mitigating liners are effective in providing better suspension, comfort, stability. A properly powered study with objective measures of socket comfort in future is warranted to determine if effective moisture mitigation at the interface can provide a better fit during activity to the amputee.

### Study limitations

This study population mostly includes the traumatic amputation patient population. Other pathologies such as diabetic or dysvascular etiology may have impaired thermoregulation^34^. So, the sweat, humidity and temperature data may differ on such patient population. Controlling against the confounding variables such as food, coffee, medication intake, physical activity, stress while driving prior to visits were beyond the study limit and may affect the data. The accuracy of the equipment recording humidity and temperature around the limb may have accuracy limits^35^.

In summary, there are limited rigorous scientific studies that clearly provide evidence-based guidelines to the prosthetist in selection of liners from numerous available options. The present study is innovative and clearly establishes the foundation of objective measures in assessing humidity and temperatures at the skin-liner interface during activity. This study provided strong evidence that relative humidity at skin-liner interface is reduced with the use of vented liner-vented socket system when compared to a similar non-vented system. Under these conditions the temperatures did not significantly change suggesting reduction of temperature alone may not play a major role in managing moisture at the liner-skin interface.

### Supplementary Information

Supplementary Information.

## Data Availability

The datasets generated and/or analyzed during the current study supporting are not publicly available to protect patient privacy but are available from the corresponding author on reasonable request.
