# Supplementary material for: Moisture mitigation using a vented liner and a vented socket system for individuals with transfemoral amputation
Source: Sci Rep. 2023 Oct 2;13:16557. doi: 10.1038/s41598-023-43572-2 (PMC10545693; doi:10.1038/s41598-023-43572-2)
Supplement: Supplementary file 1 — Supplementary Information. [file 41598_2023_43572_MOESM1_ESM.pdf]

S1. Patient population characteristics

ID	Age	Gender	Activity level	Year since amputation	Reason for amputation
AF-02	72	M	K3	12	Trauma
AF-03	35	M	K3	~1	Trauma
AF-04	52	M	K3	9	Trauma
AF-05	54	M	K3	5	Trauma
AF-06	57	M	K3	5	Trauma
AF-08	44	M	K3	7	Trauma
AF-09	41	M	K3	7	Trauma
AF-11	27	F	K3	15	Infection
AF-12	32	M	K2	~2	Trauma
